# Serotonin stimulates female preoptic area kisspeptin neurons *via* activation of type 2 serotonin receptors in mice

**DOI:** 10.3389/fendo.2023.1212854

**Published:** 2023-10-12

**Authors:** Carrie Buo, Robin J. Bearss, Alyssa G. Novak, Anna E. Anello, Jordan J. Dakin, Richard Piet

**Affiliations:** ^1^ Brain Health Research Institute and Department of Biological Sciences, Kent State University, Kent, OH, United States; ^2^ School of Biomedical Sciences, Kent State University, Kent, OH, United States

**Keywords:** hypothalamus, GnRH neuronal network, preovulatory surge, GCaMP-based calcium imaging, action potential firing

## Abstract

**Background:**

The neuroendocrine control of ovulation is orchestrated by neuronal circuits that ultimately drive the release of gonadotropin-releasing hormone (GnRH) from the hypothalamus to trigger the preovulatory surge in luteinizing hormone (LH) secretion. While estrogen feedback signals are determinant in triggering activation of GnRH neurons, through stimulation of afferent kisspeptin neurons in the rostral periventricular area of the third ventricle (RP3V^KISS1^ neurons), many neuropeptidergic and classical neurotransmitter systems have been shown to regulate the LH surge. Among these, several lines of evidence indicate that the monoamine neurotransmitter serotonin (5-HT) has an excitatory, permissive, influence over the generation of the surge, *via* activation of type 2 5-HT (5-HT_2_) receptors. The mechanisms through which this occurs, however, are not well understood. We hypothesized that 5-HT exerts its influence on the surge by stimulating RP3V^KISS1^ neurons in a 5-HT_2_ receptor-dependent manner.

**Methods:**

We tested this using kisspeptin neuron-specific calcium imaging and electrophysiology in brain slices obtained from male and female mice.

**Results:**

We show that exogenous 5-HT reversibly increases the activity of the majority of RP3V^KISS1^ neurons. This effect is more prominent in females than in males, is likely mediated directly at RP3V^KISS1^ neurons and requires activation of 5-HT_2_ receptors. The functional impact of 5-HT on RP3V^KISS1^ neurons, however, does not significantly vary during the estrous cycle.

**Conclusion:**

Taken together, these data suggest that 5-HT_2_ receptor-mediated stimulation of RP3V^KISS1^ neuron activity might be involved in mediating the influence of 5-HT on the preovulatory LH surge.

## 1 Introduction

Fertility in all mammals is ultimately controlled by gonadotropin-releasing hormone (GnRH) neurons, which drive the release of anterior pituitary gland gonadotropins luteinizing hormone (LH) and follicle-stimulating hormone. In females of spontaneously ovulating species, the rise in circulating estrogen (E) concentration stimulates the mid-cycle surge in GnRH and LH secretion that causes ovulation (1). Because GnRH neurons do not express E receptor α (ERα), the receptor required for the preovulatory surge (2–6), these cells rely on afferent ERα-expressing neurons, the GnRH neuronal network, to relay E feedback information (7). An important part of this network for mediating preovulatory E positive feedback is the preoptic area (POA) subpopulation of kisspeptin (KISS1) neurons found in two contiguous nuclei – the anteroventral periventricular nucleus and the periventricular nucleus – referred to together as the rostral periventricular area of the third ventricle (RP3V) (8, 9). RP3V^KISS1^ neurons express ERα, undergo physiological changes in response to E positive feedback, project to GnRH neurons and can drive GnRH neuron action potential firing and surge-like LH secretion [reviewed in (10–12)].

Although E positive feedback is an obligatory determinant of the preovulatory surge in spontaneous ovulators (1), many other cues regulate this neuroendocrine event through actions of neuropeptides and neurotransmitters on the GnRH neuronal network [for reviews see (7, 13–22)]. Among these, many lines of evidence indicate that the monoamine neurotransmitter serotonin (5-hydroxytryptamine, 5-HT) plays a role in the LH surge in rodents. Lesions of the dorsal raphe nucleus (DR), which comprises 5-HT-producing neurons, inhibit the LH surge (23–26). Further suggesting a role for endogenously released 5-HT, depletion of 5-HT and 5-HT receptor antagonism both suppress the LH surge (24, 27–31). Moreover, blockade of type 2 5-HT (5-HT_2_) receptors prevents the surge (32–34) while activation of 5-HT_2_ receptors is sufficient to restore the surge in animals with a DR lesion (26), indicating that 5-HT_2_ receptors might play a central role.

The mechanisms through which 5-HT might regulate the LH surge, however, are not fully understood. While evidence suggests that 5-HT neurons might influence the surge *via* indirect mechanisms (35–37), a direct influence on the GnRH neuronal network may also be at play. Indeed, 5-HT neuronal fibers are found in the RP3V (38–40) as well as in the vicinity of GnRH neurons in the POA (41), some of which originate in the DR (42). These projections might be important seeing that 5-HT release in the hypothalamus, including the POA, increases during the surge and that neurotoxic lesion of 5-HT neuron fibers in the POA prevents the surge (28, 43). At the cellular level, 5-HT can increase or decrease action potential firing and GnRH release *via* activation of multiple 5-HT receptors in immortalized GnRH neurons maintained in culture (44, 45). Some of these effects are seen in native GnRH neurons recorded in brain slices, where, curiously, most (≈75%) female GnRH neurons are inhibited by 5-HT, *via* a 5-HT_1_ receptor-mediated mechanism, whereas only a subset exhibits 5-HT_2_ receptor-mediated increases in activity (46). The impact of 5-HT neurotransmission on the LH surge, therefore, cannot be fully accounted for by effects of this neurotransmitter on GnRH neurons.

We hypothesized here that the impact of 5-HT on the LH surge is mediated, at least in part, through regulation of RP3V^KISS1^ neurons upstream of GnRH neurons. Using GCaMP-based calcium imaging and electrophysiology in brain slices, we investigated the effect of 5-HT on RP3V^KISS1^ neuron activity and the role of 5-HT_2_ receptors therein.

## 2 Materials and methods

### 2.1 Animals

Mice expressing the genetically encoded calcium indicator GCaMP6f (47) in kisspeptin neurons were generated by crossing mice that express the Cre recombinase enzyme (Cre) in kisspeptin cells (Kiss1-Cre; Jackson laboratory stock #023426) (48) with mice that express Cre-dependent GCaMP6f at the ROSA26 locus (flox-STOP-GCaMP6f; Jackson laboratory stock #028865) (49). Male and female offspring heterozygous for the Kiss1-Cre and flox-STOP-GCaMP6f alleles (Kiss1-Cre::GCaMP6f) were used in calcium imaging experiments. Female mice expressing the humanized *renilla* green fluorescent protein (hrGFP) in kisspeptin neurons (Kiss1-hrGFP; Jackson laboratory stock #023426) (50) were used in electrophysiology experiments. All experimental mice were adults (2-6 months). Female estrous cycle stage was determined by vaginal lavage (5 µL H_2_O) taken between zeitgeber time (ZT) 1 and 3 or *post-mortem* (ZT2.5-5.5). Aqueous vaginal smears were stained with methylene blue and cytology examined under light microscopy to assess estrous cycle stage (51). Mice were group-housed with littermates under controlled temperature (23 ± 2°C) and lighting (12h light/dark) conditions with *ad libitum* access to food and water. Mice were assigned to experiments based on their genotype, sex, and on their estrous cycle stage as needed. All experiments were approved by Kent State University’s Institutional Animal Care and Use Committee.

### 2.2 Fixed brain slice preparation and imaging

To visualize the distribution of GCaMP6f-expressing neurons in the RP3V of male and female mice, two male and two female (one diestrus and one proestrus) Kiss1-Cre::GCaMP6f mice were deeply anesthetized *via* intraperitoneal injection of 3 mg/mL pentobarbital before being perfused through the heart with 4% paraformaldehyde (PFA). The brains were then dissected out and placed in 4% PFA for 1 hour before being transferred to 20% sucrose in 0.1 M phosphate-buffered saline. The brains were then cut in 3 series of coronal sections (30 μm thick) using a freezing microtome. The tissue was mounted onto superfrost charged slices, air dried, and coverslipped using an aqueous mounting medium (ProLong™ Gold antifade mountant, Thermofisher). Epifluorescence images were taken in the RP3V region using a DMR microscope (Leica Microsystems) equipped with an ORCA-FLASH 4.0 V3 Digital CMOS (Hamamatsu, Japan), controlled by the MicroBrightField Neurolucida Software (MBF Bioscience).

### 2.3 Live brain slice preparation

Brain slices were prepared as previously described (52, 53). Briefly, mice were decapitated following isoflurane anesthesia, and their brains quickly removed. Coronal brain slices (200 μm thick) containing the RP3V were cut using a vibrating blade microtome (HM650V, Microm International GmbH) in an ice-cold solution containing (in mM): 87 NaCl, 2.5 KCl, 25 NaHCO_3_, 1.25 NaH_2_PO_4_, 0.5 CaCl_2_, 6 MgCl_2_, 25 glucose and 75 sucrose. Brain slices were left to incubate at 30-34°C for at least 1 hour in artificial cerebrospinal fluid (aCSF) containing (in mM): 125 NaCl, 2.5 KCl, 26 NaHCO_3_, 1.25 NaH_2_PO_4_, 2.5 CaCl_2_, 1.2 MgCl_2_ and 11 glucose. All solutions were equilibrated to pH 7.4 with a mixture of 95% O_2_/5% CO_2_. Female mice were killed between ZT2.5 and 5.5 and males between ZT2 and 4.5.

### 2.4 Calcium imaging and electrophysiology

Individual brain slices obtained from Kiss1-Cre::GCaMP6f or Kiss1-hrGFP mice were placed under an upright epifluorescence microscope (either Scientifica, UK or Prior Scientific, UK) and constantly perfused (1.5 mL/min) with warm (32-34°C) aCSF.

Variations in intracellular calcium concentration ([Ca^2+^]_i_) in RP3V^KISS1^ neurons were estimated by measuring fluorescence changes in individual GCaMP6f-expressing RP3V neurons in slices from Kiss1-Cre::GCaMP6f mice. Slices were illuminated through a 40x immersion objective, using a light-emitting diode light source (pE300^ultra^ LED; CoolLED, UK) filtered for blue light excitation (460-487 nm; Semrock, USA). Epifluorescence (emission 500-546 nm; Semrock, USA) was collected using an ORCA-FLASH 4.0 LT+ CMOS camera (Hamamatsu). LED and camera were controlled and synchronized with the μ-manager 1.4 software (54). After a ≥15-minute stabilization period in the recording chamber, a focal plane including several fluorescent cell bodies was chosen and acquisitions (100 ms light exposure at 2 Hz for 10-15 minutes) started. Low intensity LED illumination was used (≈0.1 to 0.8 mW) to minimize GCaMP6f photobleaching.

RP3V^KISS1^ neuron spontaneous action potential firing was recorded in brain slices from Kiss1-hrGFP mice. GFP-expressing RP3V neurons were visualized using brief LED illumination (excitation and emission as above) and subsequently approached with glass recording micropipettes using infrared differential interference contrast illumination. Recording micropipettes (tip resistance: 3-6 MΩ) were made with borosilicate glass (cat. #BF150-110-7.5, Sutter Instruments, USA), pulled using a Model P-1000 micropipette puller (Sutter Instruments). Action potential firing was recorded in voltage-clamp mode (no holding potential applied) in the minimally invasive cell-attached configuration (12-30 MΩ initial seal resistance). Glass micropipettes were filled with aCSF and the recording configuration was achieved by applying the lowest amount of suction required to detect spontaneous, fast, downward deflections in the current trace (spikes), which correspond to single action potentials (55). Electrical signals were recorded, filtered at 2 kHz, and digitized at 10 to 20 kHz using a double integrated patch amplifier (Sutter Instruments, USA), and acquired with the SutterPatch software (Sutter Instruments, USA).

All calcium imaging and electrophysiology experiments were performed between ZT3.5 and 10.

### 2.5 Drug applications

All drugs were dissolved to the appropriate stock concentration in water or in DMSO, aliquoted and stored at -20°C. Stock were diluted to working concentrations in aCSF prior to performing experiments. Final DMSO concentration never exceeded 0.1%. All drugs were bath-applied. Agonists were applied for one or two minutes after a ≥ three-minute baseline period in electrophysiology and in calcium imaging experiments. Antagonists and blockers were applied continuously to the slice for ≥ five minutes before a recording started and for the duration of the recording. In calcium imaging experiments, cell viability was routinely tested at the end of the experiments by applying the glutamate receptor agonists (S)-α-Amino-3-hydroxy-5-methyl-4-isoxazolepropionic acid (AMPA; 10-20 μM) or L-glutamate (100 μM), or by raising extracellular [K^+^] (+ 10 mM KCl).

AMPA (cat. #0254), the 5-HT_2_ receptor antagonists 6-[2-[4-[Bis(4-fluorophenyl)methylene]-1-piperidinyl]ethyl]-7-methyl-5H-thiazolo[3,2-a]pyrimidin-5-one (ritanserin; cat. #1955) and 1,2,3,4,10,14b-Hexahydro-2-methyldibenzo[c,f]pyrazino[1,2-a]azepine hydrochloride (mianserin; cat. #0997), the 5-HT_2A_ receptor agonist (4-Bromo-3,6-dimethoxybenzocyclobuten-1-yl)methylamine hydrobromide (TCB-2; cat. #2592), the 5-HT_2C_ receptor agonist 8,9-Dichloro-2,3,4,4a-tetrahydro-1*H*-pyrazino[1,2-a]quinoxalin-5(6*H*)-one hydrochloride (WAY161503; cat. #1801), the 5-HT_3_ receptor agonist 1-(6-Chloro-2-pyridinyl)-4-piperidinamine hydrochloride (SR57227; cat. #1205), the 5-HT_4_ receptor agonist (±)-4-Amino-5-chloro-*N*-[1-[(3R*,4S*)-3-(4-fluorophenoxy)propyl]-3-methoxy-4-piperidinyl]-2-methoxybenzamide (cisapride; cat. #1695), the 5-HT_6_ receptor agonist 3-[(-3-Fluorophenyl)sulfonyl]-*N*,*N*-dimethyl-1*H*-pyrrolo[2,3-*b*]pyridine-1-ethanamine dihydrochloride (WAY208466; cat. #3904), the 5-HT_7_ receptor agonist (2*S*)-5-(1,3,5-Trimethylpyrazol-4-yl)-2-(dimethylamino)tetralin (AS19; cat. #1968) and 5-HT hydrochloride (cat. #3547) were purchased from Tocris (Bio-techne, USA). L-glutamate (cat. #HB0383) was purchased from HelloBio (USA) and tetrodotoxin citrate (cat. #T-550) from Alomone labs (Israel).

### 2.6 Analysis

For calcium imaging, GCaMP6f fluorescence image time-series were processed in the FIJI software (56). Regions of interest (ROIs) were drawn around individual, in-focus fluorescent somata [12.1 ± 0.5 (ranging 4 to 24) and 9.2 ± 1.0 (ranging 7 to 14) ROIs per slice in females and males, respectively]. Mean fluorescence intensity within each ROI was measured in each frame. Fluorescence intensity data were analyzed using scripts written in R (http://www.r-project.org/). For each ROI, normalized fluorescence was calculated as $\frac{Ft}{F}\times 100$ , where F is the baseline fluorescence intensity calculated as the mean fluorescence intensity over a one-minute period preceding agonist applications and F_t_ is the fluorescence measured at any time point. Time-dependent bleaching was corrected by subtracting the normalized fluorescence obtained in an ROI that did not contain a fluorescent cell within the RP3V (empty ROI). This approach could not be used for recordings of the effect of AMPA, L-glutamate or KCl as these treatments induced very large increases in fluorescence that contaminated the empty ROI, thereby causing unacceptable distortions of traces upon subtraction. ROIs in which normalized fluorescence traces reversibly increased from baseline for a period of at least 2 minutes, around the time of agonist application, were recorded as being excited. For illustration and statistical comparisons, agonist effects were calculated as the mean normalized fluorescence over 30 seconds at the peak of the effect minus that over 30 seconds of baseline.

For electrophysiology, spikes were detected using the threshold crossing method. Spike time stamps were organized into ten-second bins and the mean firing rate calculated for each bin. To determine if 5-HT affected the spontaneous firing of RP3V^KISS1^ neurons, baseline firing was first measured as the mean firing rate during the two minutes preceding 5-HT application. Recordings in which the mean firing rate changed by greater than twice the standard deviation of baseline (2 × SD) during the five minutes after 5-HT first entered the bath were recorded as displaying a response to 5-HT. For statistical comparisons, firing rates were averaged over a one-minute period during baseline and at the peak of 5-HT effect.

In total, 800 GCaMP6f-expressing cells in 64 slices from 45 female mice and 55 GCaMP6f-expressing cells in 6 slices from 5 male mice were analyzed in calcium imaging experiments, while 17 GFP-expressing cells in 11 slices from 7 female mice were analyzed in electrophysiology experiments. These numbers are broken down by experiments in the results section.

Statistical analyses were performed using Prism 9.0 (GraphPad, USA). Data are reported in the text and tables as mean ± SEM, and in figures as mean ± SEM or ± 95% confidence intervals. Comparisons between two independent groups were made using the Mann-Whitney test and those between two paired groups using the paired t-test or the Wilcoxon signed rank test, as appropriate. Comparisons between multiple groups were made with the Kruskal-Wallis test with Dunn’s post-tests. Comparisons of proportions were made using Fisher’s exact tests. Differences were considered statistically significant for p< 0.05.

## 3 Results

### 3.1 5-HT stimulates RP3V^KISS1^ neuron activity in a sex-dependent, but not estrous cycle-dependent, manner

**Figure 1:**
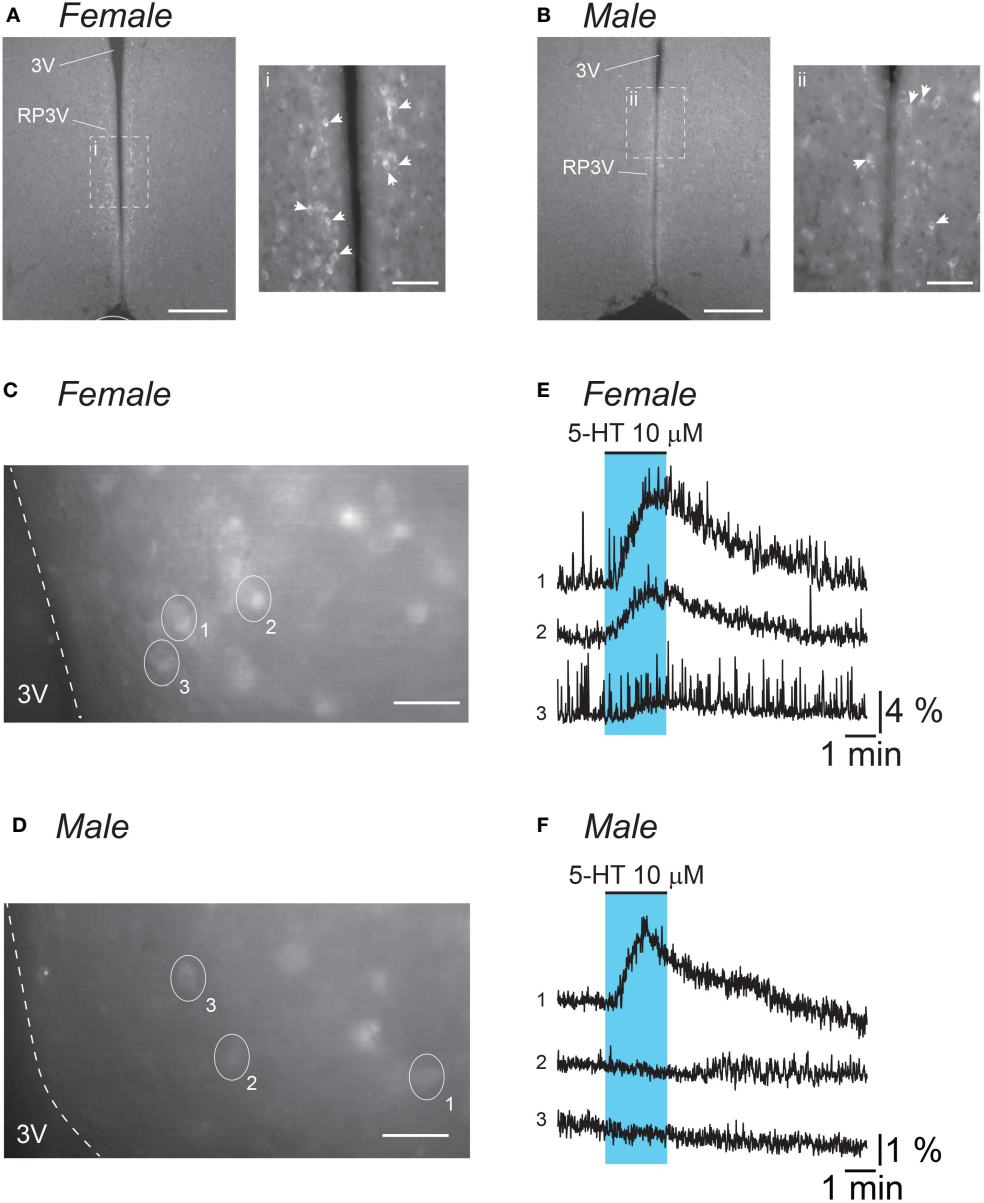
5-HT stimulates RP3V^KISS1^ neuron activity. **(A, B)** Low magnification (10X) epifluorescence images of endogenous GCaMP6f fluorescence in fixed coronal slices (30 μm thick) including the RP3V from a diestrous female **(A)** and a male **(B)** mouse. Insets i and ii are 20X magnification images of areas framed (dashed lines) in the low magnification images. Similar GCaMP6f-expressing cell distribution was observed in two different animals of each sex. White arrowheads indicate individual GCaMP6f-expressing neurons. 3V: third ventricle. Scale bars are 200 μm (50 μm in i and ii). **(C, D)** Epifluorescence images (40X magnification, average projection of 240 frames) of endogenous GCaMP6f fluorescence in acute coronal slices (200 μm thick) including the RP3V obtained from an estrous female **(C)** and a male **(D)** mouse. Ovals and numbers indicate individual regions of interest (ROIs) each including a single cell. Dashed lines delineate the border of the 3V. Scale bars 25 μm. **(E, F)** Normalized fluorescence traces for the ROIs delineated in C and D. Blue shading indicates the timing of 5-HT bath applications. Please note the difference in scale for normalized fluorescence in female **(E)** *versus* male traces **(F)**.

The effects of 5-HT on RP3V^KISS1^ neuron activity were assessed using calcium imaging in brain slices from male and female Kiss1-Cre::GCaMP6f mice (**Figures 1A, B**). In slices from female mice, two-minute bath applications of 5-HT (10 μM) resulted in transient increases in GCaMP6f fluorescence in the majority of RP3V^KISS1^ neurons (**Figures 1C, E**). In slices obtained from male Kiss1-Cre::GCaMP6f mice, 5-HT-induced increases in fluorescence could also be observed (**Figures 1D, F**), but these responses were seen much less frequently (see below).

**Figure 2:**
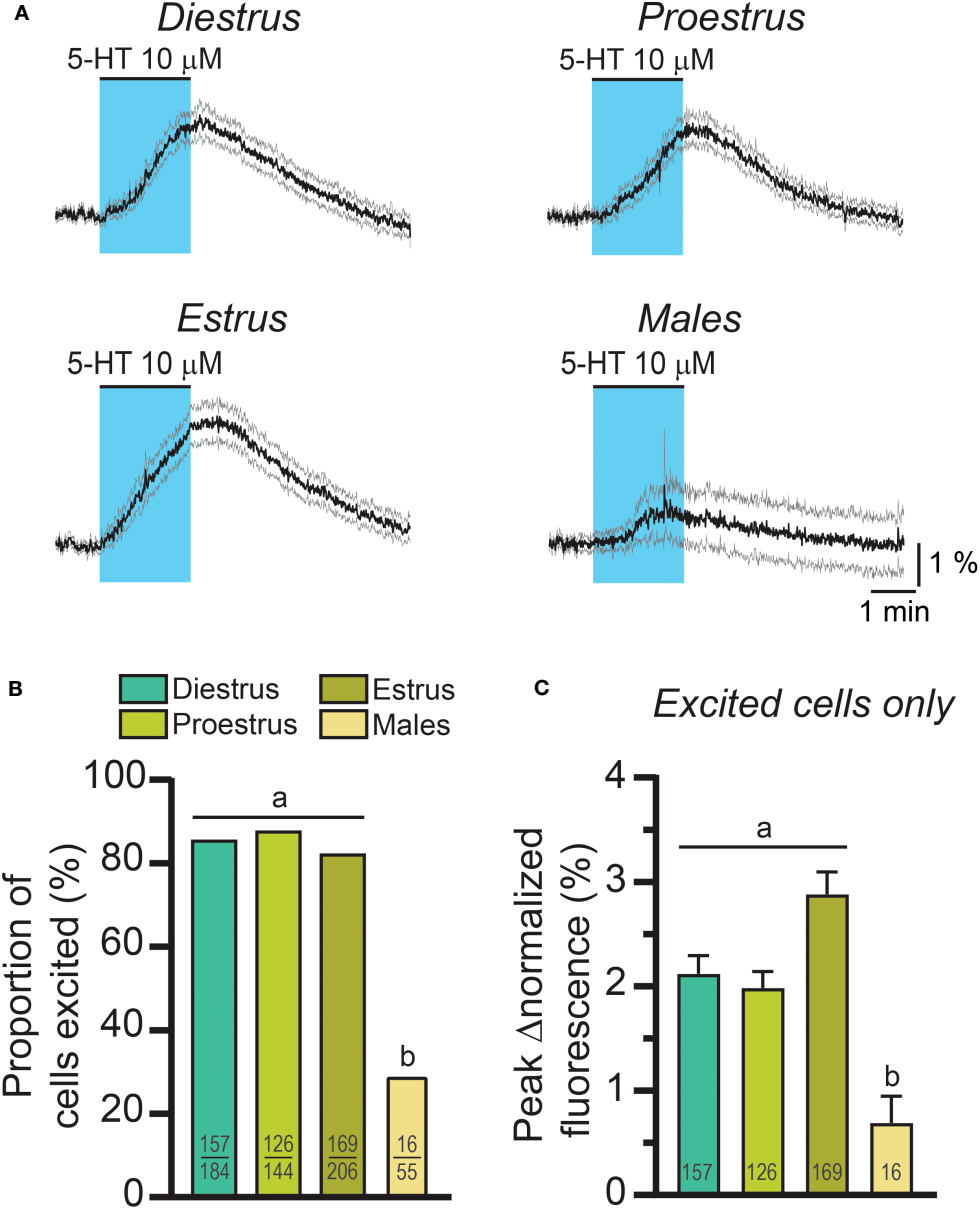
The effect of exogenous 5-HT on RP3V^KISS1^ neuron activity across the estrous cycle and in males. **(A)** 5-HT-induced changes in RP3V^KISS1^ neuron normalized fluorescence across the estrous cycle and in males. Traces are means (*black*) ± 95% confidence intervals (*grey*). **(B)** Similar proportions of RP3V^KISS1^ neurons were stimulated by 5-HT across the estrous cycle. This proportion was significantly lower in males. Numbers in bars are sample sizes (excited cells/total cells). **(C)** Peak 5-HT effect did not vary as a function of estrous cycle stage but was significantly smaller in males. Numbers in bars are sample sizes. Letters indicate results of statistical tests [Fisher’s exact tests in **(B)** Kruskal-Wallis and Dunn’s post-tests in **(C)**]. Bars with different letters are significantly different (p < 0.05; see **Table 1** for details).

Because 5-HT signaling may be involved in the generation of the LH surge, we examined RP3V^KISS1^ neuron responses to 5-HT across the estrous cycle. As illustrated in **Figure 2A**, 5-HT-induced increases in RP3V^KISS1^ neuron fluorescence could be seen in slices from diestrous, proestrous and estrous mice and were larger than those seen in slices from male mice. The proportions of RP3V^KISS1^ neurons excited by 5-HT were similar at all cycle stages (diestrus: 85.3%, proestrus: 87.5%, estrus: 82.0%; p > 0.18, Fisher’s exact tests) and were significantly higher than in males (29.1%; p < 0.001, Fisher’s exact tests; **Figure 2B** and **Table 1**). The magnitude of RP3V^KISS1^ neuron responses to 5-HT significantly varied across these groups, with male responses significantly smaller than those in females at any estrous cycle stage, but no statistical differences across the estrous cycle (**Table 1**; **Figure 2C**). Smaller responses to 5-HT in males *versus* females could not be explained by a reduced ability of male RP3V^KISS1^ neurons to mount changes in [Ca^2+^]_i_, as responses to the glutamate receptor agonist AMPA were, in fact, larger in males than in females in those slices that were tested in this manner (**Table 2**).

**Table 1:** RP3V^KISS1^ neuron responses to 5-HT across the female estrous cycle and in males.

	Diestrus	Proestrus	Estrus	Male
Total cell number	184	144	206	55
Excited cell number (proportion)	157 (85.3%)	126 (87.5%)	169 (82.0%)	16 (29.1%)
Fisher’s exact tests	p = 0.63 *versus* proestrus	p = 0.18 *versus* estrus	p = 0.41 *versus* diestrus	p < 0.001 *versus* diestrus, proestrus and estrus
Peak Δnormalized fluorescence (excited cells only)	2.12 ± 0.17%	1.98 ± 0.16%	2.88 ± 0.21%	0.69 ± 0.26%
Kruskal-Wallis test^a^	p < 0.001, K-S statistic = 22.37			
Dunn’s post-tests	p > 0.99 *versus* proestrus	p = 0.20 proestrus *versus* estrus	p = 0.12 *versus* diestrus	p < 0.001 *versus* diestrus and proestrus p < 0.001 *versus* estrus
Slice (mouse) number	14 (10)	12 (11)	17 (10)	6 (5)^b^

a the Kruskal-Wallis test was used to test for statistical differences across all 4 groups (males, di-, pro- and estrous females).

b the 16 male neurons that responded were in 4 slices from 3 different males.

**Table 2:** Response of female and male RP3V^KISS1^ neurons to stimulation by AMPA.

	Female	Male
Total cell number	299^a^	16^a^
Peak Δnormalized fluorescence (excited cells only)	18.75 ± 0.85%	27.49 ± 3.70%
Mann-Whitney test	p = 0.01, Mann-Whitney U = 1496	
Slice (mouse) number	27 (20)	4 (3)

a includes only those cells that responded to 5-HT.

Together, these observations indicate that RP3V^KISS1^ neuron responses to exogenous 5-HT are – to some extent – sex-dependent, being more prominent in females. Female responses, however, are not affected by the estrous cycle. The remainder of the study was conducted in brain slices from females.

### 3.2 5-HT increases female RP3V^KISS1^ neuron action potential firing

**Figure 3:**
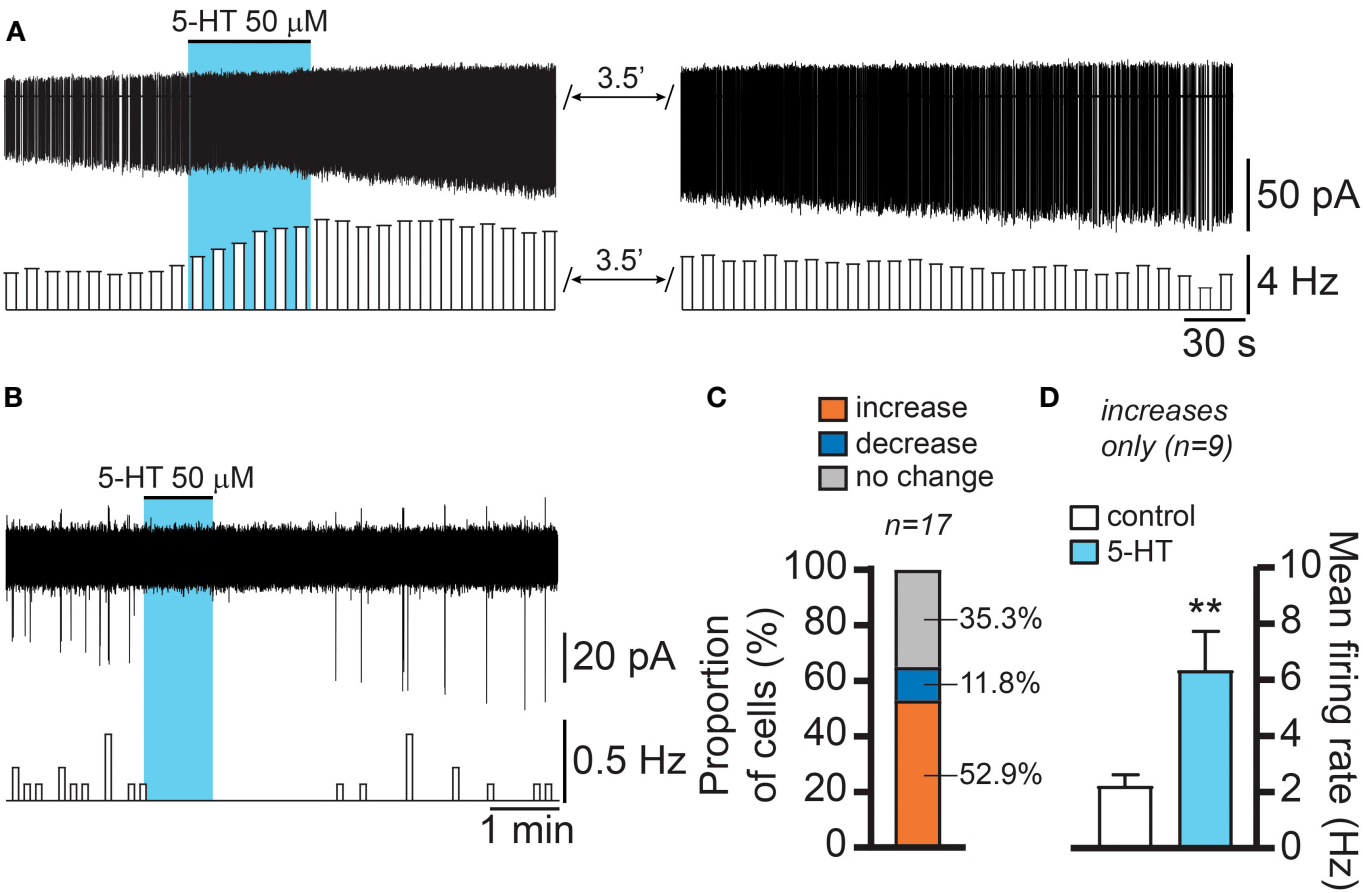
5-HT effects on female RP3V^KISS1^ neuron action potential firing. **(A, B)** Example traces and corresponding rate-meters illustrating the excitatory **(A)** and inhibitory **(B)** effects of 5-HT on female RP3V^KISS1^ neuron firing. Blue shading indicates the timing of 5-HT bath applications. **(C)** Proportions of RP3V^KISS1^ neurons that showed increased, decreased or no change in firing in response to 5-HT. **(D)** In those cells that were excited by 5-HT, the increase in firing was statistically significant. **p < 0.01 paired t-test. Numbers above bars are sample sizes.

We then investigated if the effect of 5-HT was associated with changes in electrical activity in RP3V^KISS1^ neurons. In slices from female Kiss1-hrGFP mice, we recorded individual RP3V GFP-expressing neurons in the cell-attached configuration. Bath applications of 5-HT (one minute, 50 μM) increased action potential firing in just over half (52.9%) of RP3V^KISS1^ neurons (9 out of 17 neurons in 8 slices from 5 mice [1 di- and 4 estrus]; **Figures 3A, C**), decreased it in a few cells (2 cells [11.8% of total] in 2 slices from 2 mice [1 di- and 1 estrus]; **Figures 3B, C**), or did not affect firing (6 neurons [35.3% of total] in 5 slices from 4 mice [1 pro-, 1 di- and 2 estrus]). In those RP3V^KISS1^ neurons that were stimulated by 5-HT, action potential firing increased from 2.20 ± 0.42 to 6.33 ± 1.40 Hz (p < 0.01, t = 3.66, df = 8, paired t-test; **Figure 3D**). In the two cells inhibited by 5-HT, spontaneous firing (0.73 and 0.12 Hz) was transiently silenced in both cases.

These observations indicate that 5-HT primarily stimulates RP3V^KISS1^ neuron action potential firing. While 5-HT could also suppress firing in RP3V^KISS1^ neurons, this was seen much less frequently.

### 3.3 The effect of 5-HT on female RP3V^KISS1^ neuron [Ca^2+^]_i_ is direct

**Figure 4:**
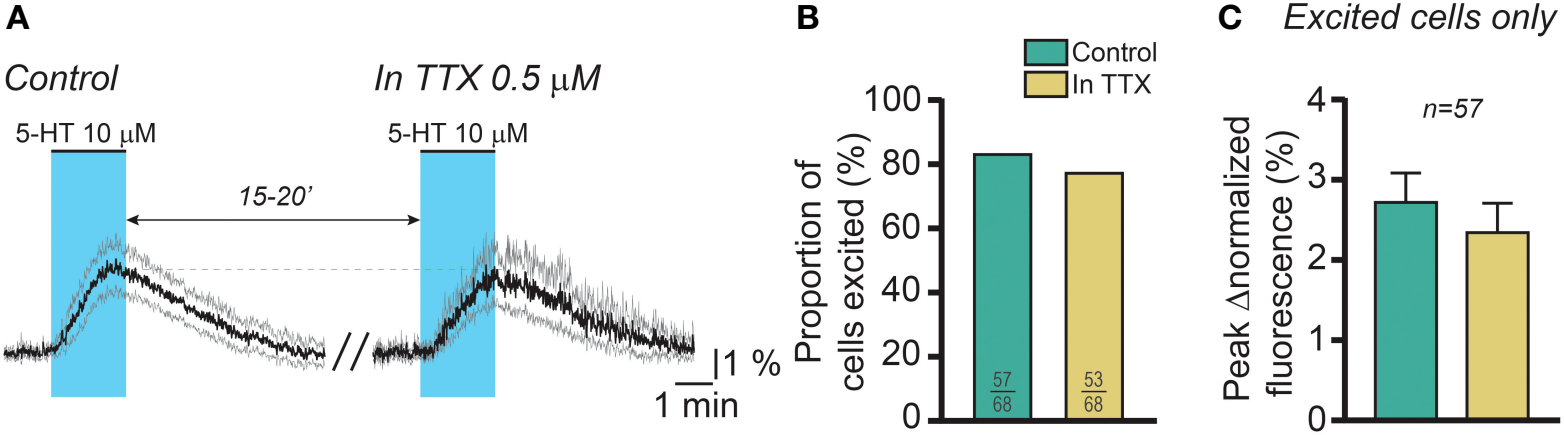
The effect of 5-HT on female RP3V^KISS1^ neuron activity is direct. **(A)** 5-HT was applied twice to brain slices from female mice, at 15-to-20-minute intervals. The first application (control; *left*) was carried out in the absence of drugs whereas the second was conducted in the continuous presence of tetrodotoxin (TTX; *right*) to block action potentials. Traces are means (*black*) ± 95% confidence intervals (*grey*) and include those RP3V^KISS1^ neurons that were stimulated by 5-HT upon the first application (n = 57). Blue shading indicates the timing of 5-HT bath applications. **(B)** Similar proportions of RP3V^KISS1^ neurons were stimulated by 5-HT in the presence and in the absence of TTX. Numbers in bars are sample sizes (excited cells/total cells). **(C)** The peak magnitude of the 5-HT effect was similar in the presence and in the absence of TTX. Only RP3V^KISS1^ neurons excited by 5-HT upon the first application were included in this analysis. The number above the bars is the sample size.

We next tested whether the effect of 5-HT is direct or mediated through the release of an intermediary factor by cells within the slice. To do this, we used a protocol in which slices from Kiss1-Cre::GCaMP6f females were exposed to 5-HT (two minutes, 10 μM) twice at a 15- to 20-minute interval, the second 5-HT application being carried out in the presence of the voltage-gated sodium channel inhibitor tetrodotoxin (TTX; 0.5 μM) to block electrical activity in the slice. This concentration of TTX is sufficient to inhibit action potential generation in *ex vivo* preparations (57–59). As illustrated in **Figure 4A**, 5-HT-induced increases in RP3V^KISS1^ neuron fluorescence persisted in the presence of TTX. In control conditions, 57 out of 68 cells (83.8%) were stimulated by 5-HT. In the presence of TTX, 53 of these neurons (77.9% of the total; p = 0.51, Fisher’s exact test; **Figure 4B**) displayed 5-HT-induced increases in normalized fluorescence. On average, 5-HT increased RP3V^KISS1^ neuron normalized fluorescence to a similar extent whether in the absence (2.72 ± 0.37%) or in the presence of TTX (2.34 ± 0.37%; n = 57 from 7 slices in 4 mice [2 pro-, 1 di- and 1 estrus]; p = 0.24, Wilcoxon test; **Figure 4C**).

This finding indicates that the effect of 5-HT on RP3V^KISS1^ neuron activity is likely direct.

### 3.4 Activation of 5-HT_2_ receptors mimics the effect of 5-HT in female RP3V^KISS1^ neurons

**Figure 5:**
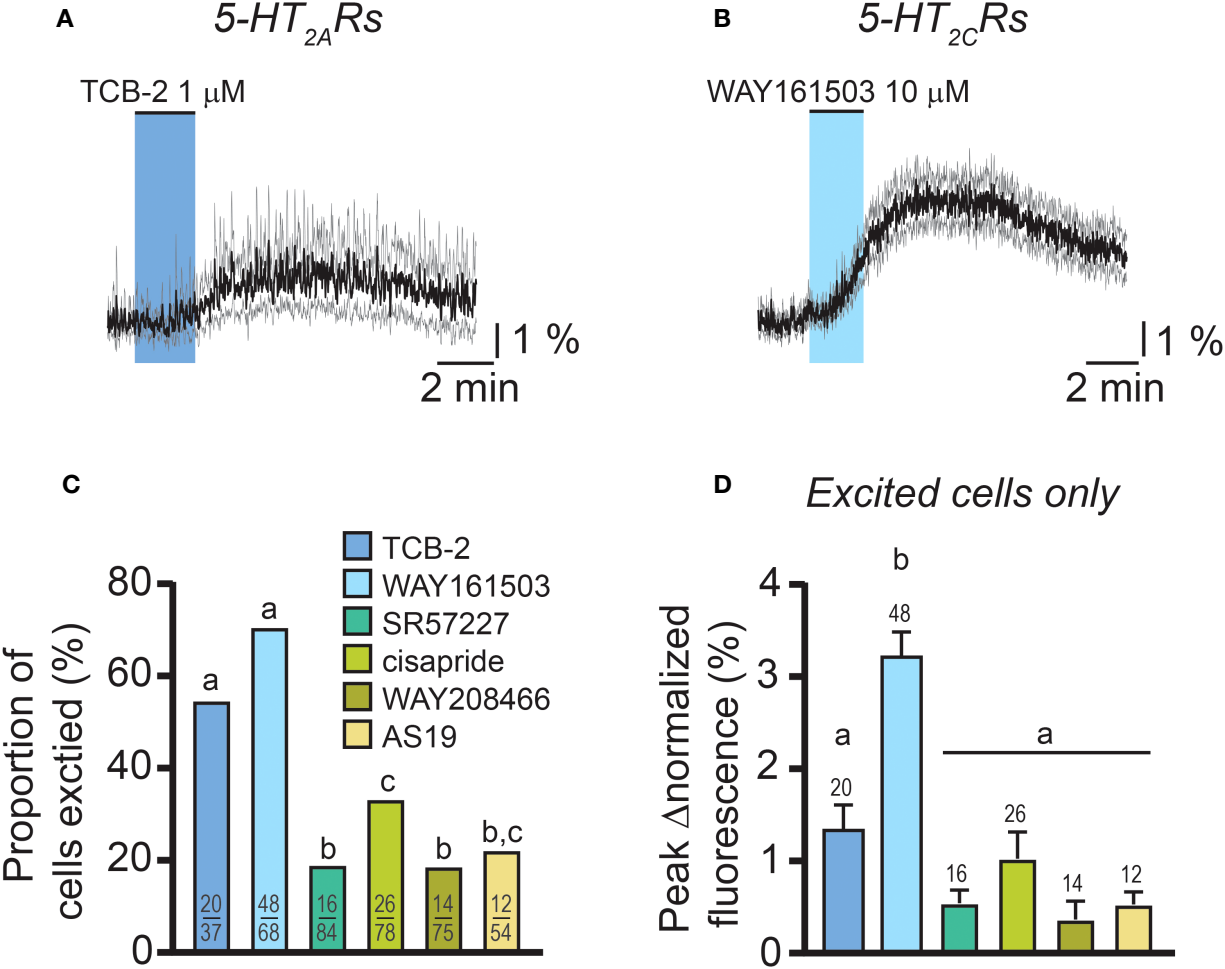
Responses of female RP3V^KISS1^ neurons to excitatory 5-HT receptor agonists. **(A, B)** Average responses of female RP3V^KISS1^ neurons stimulated by 5-HT_2A_ **(A)** and 5-HT_2C_ **(B)** receptor agonists. Colored shading indicates the timing of agonist applications. Traces are means (*black*) ± 95% confidence intervals (*grey*). **(C)** Proportions of female RP3V^KISS1^ neurons stimulated by 5-HT receptor agonists. Numbers in bars are sample sizes (excited cells/total cells). **(D)** Magnitude of the effect of different 5-HT receptor agonists. Only RP3V^KISS1^ neurons that responded to agonists were included in this analysis. Numbers above bars are sample sizes. Letters above bars represent the results of statistical tests [Fisher’s exact tests in **(C)** and Kruskal-Wallis and Dunn’s post-tests in **(D)**]. Bars that do not share a letter are significantly different (p < 0.05, see **Table 3** for details).

Because female RP3V^KISS1^ neurons express genes for multiple stimulatory 5-HT receptor subtypes, including *htr2a*, *htr2c*, *htr3a*, *htr4*, *htr6* and *htr7* (60), we next examined if activating these 5-HT receptors could increase [Ca^2+^]_i_ in female RP3V^KISS1^ neurons. Increases in RP3V^KISS1^ neuron activity could be seen in response to bath-applications of the agonists TCB-2 (1 μM; 5-HT_2A_ receptors; 2 di-, 1 pro- and 1 estrous mice), WAY161503 (10 μM; 5-HT_2C_ receptors; 3 diestrous mice), SR57227 (1 μM; 5-HT_3_ receptors; 4 diestrous mice), cisapride (1 μM; 5-HT_4_ receptors; 3 diestrous mice), WAY208466 (10 μM; 5-HT_6_ receptors; 3 diestrous mice) and AS19 (10 μM; 5-HT_7_ receptors; 1 di-, 3 pro- and 1 estrous mice) (**Figures 5A, B** and **Table 3**). However, activating 5-HT_2A_ and 5-HT_2C_ receptors resulted in greater proportions of stimulated RP3V^KISS1^ neurons (≈55-70%) than activating other receptor subtypes (**Figure 5C** and **Table 3**). In addition, activating 5-HT_2C_ receptors resulted in significantly larger responses than activating other 5-HT receptors, whereas responses to 5-HT_2A_ receptor activation were rather moderate in magnitude (**Figure 5D** and **Table 3**).

**Table 3:** RP3V^KISS1^ neuron excitatory responses to 5-HT receptor agonists in female mice.

	TCB-2	WAY161503	SR57227	Cisapride	WAY208466	AS19
Total cell number	37	68	84	78	75	54
Excited cell number (proportion)	20 (54.1%)	48 (70.6%)	16 (19.0%)	26 (33.3%)	14 (18.7%)	12 (22.2%)
Fisher’s exact tests	p < 0.001 *versus* SR57227 and WAY208466	p < 0.001 *versus* SR57227, cisapride, WAY208466 and AS19	p < 0.05 *versus* cisapride	p < 0.05 *versus* TCB-2	p < 0.05 *versus* cisapride	p < 0.01 *versus* TCB-2
Peak Δnormalized fluorescence (excited cells only)	1.33 ± 0.28%	3.21 ± 0.28%	0.54 ± 0.14%	1.03 ± 0.29%	0.37 ± 0.20%	0.53 ± 0.14%
Kruskal-Wallis test^a^	p < 0.001, K-S statistic = 62.09					
Dunn’s post-tests	/	p < 0.01 *versus* TCB-2	p < 0.001 *versus* WAY161503	p < 0.001 *versus* WAY161503	p < 0.001 *versus* WAY161503	p < 0.001 *versus* WAY161503
Slice (mouse) number	5 (4)	5(3)	7 (4)^b^	6(3)	5(3)^b^	5(5)^b^

a the Kruskal-Wallis test was used to test for statistical differences across the effect of all 6 agonists (TCB-2, WAY161503, SR57227, cisapride, WAY208466 and AS19).

b RP3V^KISS1^ cells showing excitatory responses to SR57227, WAY208466 and to AS19 were found in 6 slices from 4 mice, in 4 slices from 3 mice and in 3 slices from 3 mice, respectively.

Taken together, these observations indicate that the female RP3V^KISS1^ neuron population expresses multiple functional excitatory 5-HT receptor subtypes. However, of these, activation of 5-HT_2_ receptors, in particular 5-HT_2C_, most closely mimics the effect of 5-HT on female RP3V^KISS1^ neurons.

### 3.5 5-HT stimulates female RP3V^KISS1^ neuron activity *via* activation of 5-HT_2_ receptors

**Figure 6:**
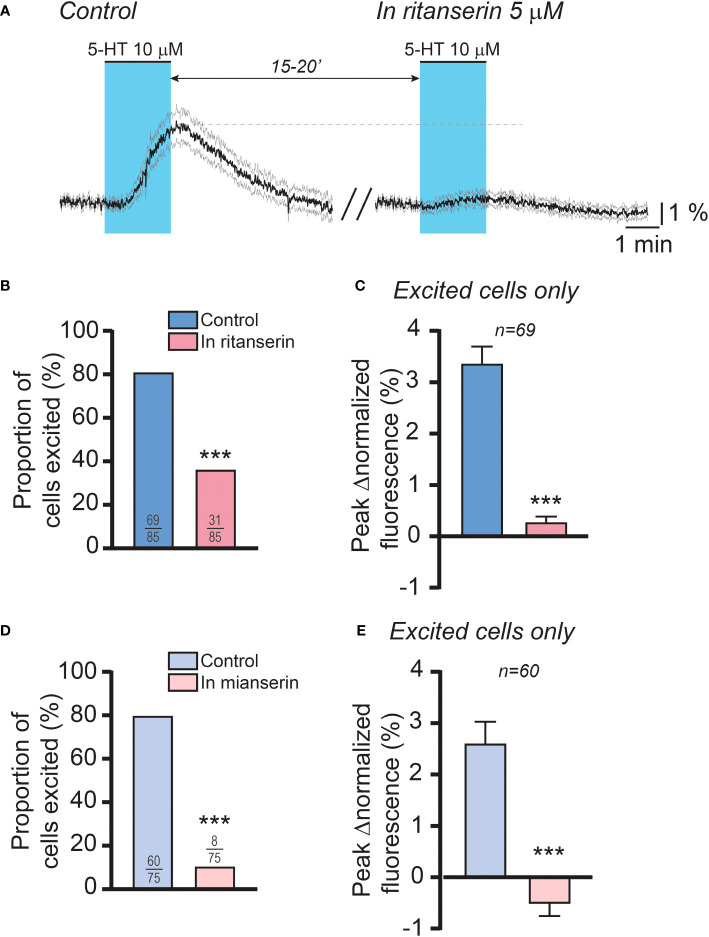
5-HT2 receptors mediate the effect of 5-HT on female RP3V^KISS1^ neuron activity. **(A)** 5-HT was applied twice to female brain slices, at 15-to-20-minute intervals. The first application (control; *left*) was carried out in the absence of drugs whereas the second was conducted in the continuous presence of ritanserin (*right*), a 5-HT2 receptor antagonist. Light blue shading indicates the timing of 5-HT bath applications. Traces are means (*black*) ± 95% confidence intervals (*grey*). **(B)** Proportions of RP3V^KISS1^ neurons stimulated by 5-HT significantly decreased in the presence of ritanserin. ***p < 0.001, Fisher’s exact test. Numbers in bars are sample sizes (excited cells/total cells). **(C)** Ritanserin significantly decreased the peak magnitude of the 5-HT effect. ***p < 0.001, Wilcoxon signed rank test. Number above bars is the sample size. **(D)** Mianserin significantly reduced the proportions of RP3V^KISS1^ neurons stimulated by 5-HT. ***p < 0.001, Fisher’s exact test. Numbers in bars are sample sizes (excited cells/total cells). **(E)** The peak magnitude of the 5-HT effect was significantly suppressed by mianserin. ***p < 0.001, Wilcoxon signed rank test. Number above bars is the sample size. Only RP3V^KISS1^ neurons that responded to 5-HT upon the first bath application were included in the analyses displayed in **(C, E)**.

Based on these findings, we then sought to determine the role of 5-HT_2_ receptors in mediating the effect of 5-HT on RP3V^KISS1^ neuron activity. Using a dual 5-HT application protocol like that described above, we tested the impact of 5-HT_2_ receptor antagonists on RP3V^KISS1^ neuron responses to 5-HT. As illustrated in **Figure 6A**, the antagonist ritanserin (5 μM), which prevents the LH surge in rats (34), almost completely blocked the effect of 5-HT in RP3V^KISS1^ neurons. 69 out of 85 cells (81.2%) were stimulated by 5-HT in control conditions. In the presence of ritanserin, 31 of these (36.5% of the total; p < 0.001, Fisher’s exact test) had 5-HT-induced increases in normalized fluorescence (**Figure 6B**). On average, 5-HT-induced changes in RP3V^KISS1^ neuron normalized fluorescence were substantially reduced by ritanserin (0.29 ± 0.10% *versus* 3.37 ± 0.32% in the absence of antagonist; n = 69 in 5 slices from 5 mice [2 pro-, 2 di- and 1 estrus]; p < 0.001, sum of signed ranks (W) = -2387, Wilcoxon test; **Figure 6C**). The same held true when only those cells that were excited by 5-HT in the presence of ritanserin were considered (**Table 4**). Similar results were obtained with the antagonist mianserin (10 μM). In its presence, only 8 out of 75 cells (10.7% *versus* 80.0% [60 out of 75] in control; p < 0.001, Fisher’s exact test; **Figure 6D**) were excited by 5-HT. Moreover, 5-HT-induced increases in normalized fluorescence were significantly suppressed by mianserin (-0.53 ± 0.22% *versus* 2.61 ± 0.42% in the absence of antagonist; n = 60 in 6 slices from 5 mice [1 pro-, 2 di- and 2 estrus]; p < 0.001, sum of signed ranks (W) = -1718, Wilcoxon test; **Figure 6E**), even when only cells stimulated by 5-HT in the presence of the antagonist were considered (**Table 4**).

**Table 4:** Excitatory effects of 5-HT in the presence of 5-HT_2_ receptor antagonists ritanserin or mianserin.

	Control (Peak Δnormalized fluorescence)	In antagonist (Peak Δnormalized fluorescence)	number of cells (slices; mice)	Wilcoxon signed rank tests
Ritanserin	2.99 ± 0.38%	0.55 ± 0.10%	31 (3; 3^a^)	p < 0.001, sum of signed ranks W = -490
Mianserin	5.31 ± 1.62%	0.03 ± 0.66%	8 (5; 4^a^)	p < 0.05, sum of signed ranks W = -32

a ritanserin: 2 pro- and 1 estrous mice; mianserin: 1 pro-, 1 di- and 2 estrous mice.

Together with the results obtained using 5-HT receptor agonists, these data indicate that the effect of 5-HT on RP3V^KISS1^ neurons is – for the most part – mediated by 5-HT_2_ receptors, likely through the combined effect of 5-HT_2A_ and 5-HT_2C_ receptor activation.

## 4 Discussion

We report here using GCaMP-based [Ca^2+^]_i_ imaging and electrophysiology that exogenous 5-HT stimulates the activity of a majority of RP3V^KISS1^ neurons in female mice. This effect is observed in higher proportions of RP3V^KISS1^ neurons – and with larger magnitudes – in females than in males. In females, however, the effect of 5-HT does not significantly vary between estrous cycle stages. Lastly, we find that 5-HT-induced excitations are likely mediated directly at RP3V^KISS1^ neurons and that these effects require activation of 5-HT_2_ receptors. Together, these observations suggest that stimulation of RP3V^KISS1^ neurons might be a mechanism through which 5-HT influences the LH surge in rodents.

A large proportion of female RP3V^KISS1^ neurons (> 80%) exhibited increases in [Ca^2+^]_i_ in response to 5-HT in brain slices from Kiss1-Cre::GCaMP6f mice. In slices from Kiss1-hrGFP mice, 5-HT caused an increase in action potential firing in > 50% of RP3V^KISS1^ neurons. Increases in [Ca^2+^]_i_ induced by 5-HT likely result from opening of voltage-gated Ca^2+^ channels in response to action potential firing and/or to subthreshold membrane depolarization, from G-protein-dependent Ca^2+^ release from internal stores, or from a combination thereof (10, 61). As the cell-attached patch-clamp recording configuration does not give access to subthreshold membrane potential fluctuations – nor to changes in [Ca^2+^]_i_ – the proportions of neurons that responded to 5-HT applications with elevations in [Ca^2+^]_i_ and in firing rates cannot be directly compared. Interestingly, 5-HT suppressed firing in a small subset (≈ 10%) of RP3V^KISS1^ neurons in electrophysiology experiments. It is possible that such responses were not seen with our [Ca^2+^]_i_ imaging because, at least under our recording conditions, this approach does not effectively resolve inhibitions in those cells that display low resting activity levels (10, 61). Nevertheless, our observation that 5-HT stimulated activity in a majority of RP3V^KISS1^ neurons is in line with a recent report that 5-HT increases firing in arcuate kisspeptin (ARC^KISS1^) neurons (62), suggesting that stimulation by 5-HT is a common feature of hypothalamic kisspeptin neurons. Somewhat contrastingly, only a small proportion of adult female GnRH neurons (< 40%) are stimulated by 5-HT, whereas most (≈ 75%) are inhibited (46).

The stimulatory effect of 5-HT on RP3V^KISS1^ neuron [Ca^2+^]_i_ was largely resistant to blockade of action potential firing, suggesting that responses to 5-HT were mediated primarily through action potential-independent VGCC opening and/or mobilization of intracellular Ca^2+^ stores. This also reveals that RP3V^KISS1^ neuron [Ca^2+^]_i_ responses to 5-HT were independent on electrical activity within the brain slice and, therefore, resulted from direct 5-HT actions at the kisspeptin neurons. Moreover, RP3V^KISS1^ neurons displayed excitatory responses to 5-HT_2A_, 5-HT_2C_, 5-HT_3_, 5-HT_4_, 5-HT_6_ and 5-HT_7_ receptor activation. This reveals that RP3V^KISS1^ neurons may express multiple functional 5-HT receptor subtypes. However, activation of 5-HT_2C_ and – to a lesser extent – 5-HT_2A_ receptors most closely mimicked the stimulatory effect of 5-HT. Importantly, the stimulatory effect of 5-HT was blocked by the 5-HT_2_ receptor antagonists ritanserin and mianserin. Together, these observations indicate that 5-HT stimulates RP3V^KISS1^ neuron activity by acting at 5-HT_2_ receptors, including 5-HT_2A_ and 5-HT_2C_ receptors. 5-HT is also reported to stimulate the activity of ARC^KISS1^ neurons, but this is mostly mediated by 5-HT_4_ receptors (62). The two main populations of hypothalamic kisspeptin neurons may, therefore, have different functional 5-HT receptor make-ups. Nevertheless, our observations in RP3V^KISS1^ neurons are in line with previous reports that ritanserin prevents the LH surge in rats (33, 34), and that activation of 5-HT_2A_ and 5-HT_2C_ receptors restores the LH surge in DR-lesioned female rats (26). Seeing the pattern and pharmacology of RP3V^KISS1^ neuron responses to 5-HT – and, as discussed above, that 5-HT may primarily inhibit female GnRH neurons (46) – we propose that the stimulatory effect of 5-HT we report here might be involved in mediating the previously reported excitatory influence of 5-HT neurotransmission on the LH surge (27–30, 32–34).

5-HT responses were found in a substantially and significantly lower proportion of RP3V^KISS1^ neurons in males (≈ 35%) than in females. Further, those male RP3V^KISS1^ neurons that were stimulated by 5-HT displayed much smaller responses than their female counterparts. Although we cannot fully rule out that a proportion of the GCaMP6f-expressing cells that we recorded in slices from male mice were RP3V neurons that do not, in fact, express *Kiss1* (10, 63–65), this suggests that 5-HT excitatory effects in RP3V^KISS1^ neurons display some degree of sex-dependence. As the LH surge mechanism, including many aspects of the RP3V^KISS1^ → GnRH neural circuit, is sexually differentiated in rodents (66–69), this observation suggests that 5-HT signaling in RP3V^KISS1^ neurons might be part of the mechanism activating these cells for the surge. On the other hand, we find that the impact of 5-HT on RP3V^KISS1^ neuron activity did not significantly change between estrous cycle stages, with similar proportions of neurons stimulated and similar response magnitude. This suggests that regulation of 5-HT_2_ receptor function in RP3V^KISS1^ neurons might not be a mechanism through which these cells are specifically activated for the proestrous surge. Rather, this suggests that 5-HT might have a permissive effect on RP3V^KISS1^ neuron activity as it does on the surge *in vivo* (15). On the other hand, 5-HT content and turnover in the hypothalamus, including the POA, might fluctuate around the time of the proestrous surge in a diurnal manner (28, 70, 71). Whether 5-HT release in the vicinity of RP3V^KISS1^ neurons fluctuates in a time- and estrous cycle-dependent manner is unknown but would be anticipated to affect RP3V^KISS1^ neuron activity.

5-HT-containing fibers are detected within the RP3V in male and female rats (38–40), but it is unknown whether these fibers are in the vicinity of kisspeptin neurons. The origin of these fibers is also unknown. They may originate in the DR as lesions of this area, more so than lesions of the medial raphe nucleus, prevent the LH surge (23–26). Curiously, electrical stimulation of the DR fails at altering the LH surge in proestrous rats (24), whereas this manipulation can evoke LH secretion in E-replaced OVX rats (72) and result in a subtle prolongation of LH secretion evoked by stimulation of the POA (73). DR neurons project directly to POA GnRH neurons (42); whether they project to RP3V^KISS1^ neurons will need to be established by tract-tracing experiments. It should be noted that 5-HT transporter-immunoreactive fibers are seen in close apposition to ARC^KISS1^ neurons (62). As ARC^KISS1^ neurons might be involved in regulating the LH surge (74–78), it is possible that these fibers contribute to the influence of 5-HT on the surge. As discussed above, the pharmacology of ARC^KISS1^ neuron responses to 5-HT does not match that of the influence of 5-HT on the surge, however.

Lastly, the type of information that 5-HT neurons relay to the GnRH neuronal network to regulate the surge is unknown. 5-HT may mediate the permissive effect of the metabolic hormone leptin on the hypothalamic-pituitary-gonadal axis (79) and, indeed, the DR is involved in many aspects of energy balance (80). Additionally, 5-HT signaling may be involved in regulating circadian rhythms and responses to stressors (81–83), which also affect LH secretion and the surge (20, 21). Further studies will be required to determine the precise contribution of changes in 5-HT neurotransmission in regulating the surge and whether this involves RP3V^KISS1^ neurons.

In conclusion, the findings reported here regarding the stimulatory effect of 5-HT signaling on RP3V^KISS1^ neuron activity contribute to our understanding of the brain circuits that control ovulation in females.

## Data availability statement

The raw data supporting the conclusions of this article will be made available by the authors, without undue reservation.

## Ethics statement

The animal study was approved by Kent State University Institutional Animal Care and Use Committee. The study was conducted in accordance with the local legislation and institutional requirements.

## Author contributions

RP designed the research. CB, RB, AN, AA and JD carried out the experiments. CB, RB, AN, AA, and RP analyzed the data. RP wrote the manuscript. All authors contributed to the article and approved the submitted version.
